# Isolation and Identification of *Staphylococcus aureus* from Milk and Milk Products, Associated Factors for Contamination, and Their Antibiogram in Holeta, Central Ethiopia

**DOI:** 10.1155/2022/6544705

**Published:** 2022-05-06

**Authors:** Endrias Zewdu Gebremedhin, Addisu Bedashu Ararso, Bizunesh Mideksa Borana, Kebede Abdisa Kelbesa, Nega Desalegn Tadese, Lencho Megersa Marami, Edilu Jorga Sarba

**Affiliations:** ^1^Department of Veterinary Sciences, College of Agriculture and Veterinary Science, Ambo; University, P.O. Box 19, Ambo, Ethiopia; ^2^Department of Microbiology, Holeta Polytechnic College, P.O. Box 11, Oromia, Ethiopia; ^3^Department of Veterinary Laboratory Technology, College of Agriculture and Veterinary Science, Ambo University, P.O. Box 19, Ambo, Ethiopia

## Abstract

*Staphylococcus aureus* is a pathogenic bacterium-contaminating milk and milk products causing food poisoning primarily due to its enterotoxins. The study aimed at estimating the prevalence of *S. aureus* in milk and milk products, assessing potential risk factors for contamination, and determining the load and the antimicrobial susceptibility profile of the isolates. A cross-sectional study design was employed to collect a total of 486 samples, comprising 383 raw milk, 47 bulk tank milk, 29 curd milk (Ergo), and 28 Ethiopian cottage cheese (Ayib) samples. Enumeration, isolation, and identification of *S. aureus* were carried out following standard microbiological techniques. Antibiogram was performed using 12 antimicrobials following the Kirby–Bauer disc diffusion method. Logistic regression analyses were used to assess the association between the occurrence of *S. aureus* in milk and milk products and potential risk factors. The overall prevalence of *S. aureus* was 10.69% (52/486) [95% confidence interval (CI):8.09–13.79%]. The prevalence of *S. aureus* in raw milk, curd milk, bulk tanks at the farm, bulk tanks at milk collection facilities, and cottage cheese was 8.64%, 24.14%, 14.73%, 23.08%, and 14.29%, respectively. The rate of isolation of *S. aureus* was significantly high in curd milk than in other types of samples (*P* = 0.010). The study revealed that teat washing (OR: 4.93, 95% CI: 2.06–11.81), use of towel (OR: 12.13, 95% CI: 3.74–39.29), and tick infestations (OR: 4.31, 95% CI: 1.28–14.44) were risk factors associated with the occurrence of *S. aureus* in milk. About 48.39% of the milk samples assessed had the *S. aureus* count higher than 10^5^ CFU/ml. The highest rate of resistance was observed to ampicillin (95%), amoxicillin (95%), oxacillin (87.5%), and cefotaxime (80%). All isolates are resistant to at least two classes of antimicrobial drugs, while 65.0% of the isolates were found to be multidrug-resistant. The moderate prevalence, high load, and antimicrobial resistance of *S. aureus* indicate the higher public health risk due to the widespread consumption of raw milk in the area. Good hygienic practices, regular surveillance of antimicrobial resistance, and prudent use of drugs are suggested.

## 1. Introduction

In developing countries, food-borne infections constitute the major cause of sickness and death. Food-related illnesses are caused by changes in eating patterns, mass catering, improper food storage conditions, and inadequate hygienic procedures, which result in 600 million morbidities and 33 million deaths worldwide [1]. *S. aureus* case fatality rates are 0.03% [2]. This is especially true in developing countries like Ethiopia, where the production of milk and various dairy products often occurs under unsanitary conditions and the consumption of raw milk is common [3].

Staphylococcal food poisoning (SFP) is one of the most prevalent food-borne diseases worldwide, second only to salmonellosis in terms of prevalence [4, 5]. *Staphylococci cause food contamination, decomposition, and a decline in food quality and shelf life, as well as food poisoning through the formation of fatal enterotoxins* [6]. The frequency of *Staphylococcus* varies between farm and dairy products due to storage, handling, use of unsanitary utensils, and milking circumstances, as well as genetic heterogeneity in disease resistance among the breeds maintained in the system [7, 8].

*S. aureus* contamination of dairy cows and raw milk is still a problem in the dairy food industry. The multiplicity of food-borne disease outbreaks linked to tainted dairy products demonstrates *S*. *aureus*' public health importance [9]. Dairy animals are the most likely source of contamination of raw milk by *Staphylococcus aureus.* Contamination of dairy herds and raw milk by *S. aureus* remains an important issue in dairy food production. *S. aureus* public health significance is evidenced by the plethora of food-borne disease outbreaks resulting from contaminated dairy products. Dairy animals are probably the main source of contamination of raw milk with *Staphylococcus aureus* [10].

The spread of antimicrobial-resistant staphylococci, which could be owing to indiscriminate antimicrobial usage by healthcare providers, untrained practitioners, and medication consumers, poses a problem for both human and animal health experts [11]. The susceptibility of *S. aureus* to penicillin G and tetracycline is very low due to the regular use of these drugs for the treatment of cows that may result in the spread of resistant strains in most areas of Ethiopia [12].

Studying epidemiology and antimicrobial resistance of *S. aureus* in milk and its products in Ethiopia is crucial for developing ways to reduce the risk of food-borne disease and antimicrobial resistance. In Ethiopia, improper handling methods of milk and milk products and a low level of food handler education and attitudes contribute to food contamination. In some locations of Ethiopia, such as in Holeta [13], Hawasa [14], in Adama [15], and in and around Addis Ababa [16], investigations on *S. aureus* isolation, identification, and antimicrobial susceptibility tests have only focused on animal health issues. However, data on food poisoning caused by *S. aureus*, as well as its load in milk and its products across the dairy production chain and drug resistance trends, are scarce. This study was carried out to estimate the prevalence and associated risk factors of *S. aureus* in milk and milk products and determine the load and antibiogram of *S. aureus* isolates in Holeta town, central Ethiopia.

## 2. Methods

### 2.1. Study Area

**Figure 1:**
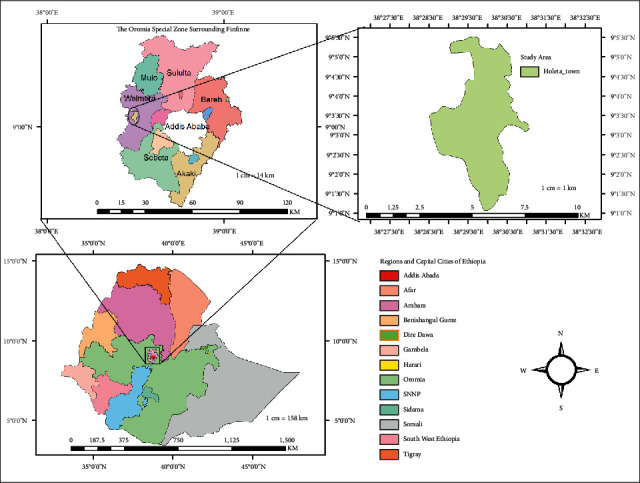
Map of Holeta town.

The study was conducted in Holeta town, Oromia regional state, Ethiopia (Figure 1). Holeta town is located in the special zone surrounding Finfinne, at a distance of 44 km from Finfinne in the western direction. The 2007 national census reported a total population for Holeta of 25,593, of whom 12,605 were men and 12,988 were women [17]. The town has a latitude of 9°3′N and a longitude of 38°30′E and an altitude of 2391 meters above sea level. The area has mild subtropical weather with a minimum and maximum annual temperature of 6.3°C and 22.1°C, respectively, which is on average 14.5°C. The area also experiences a bimodal rainfall pattern, with a long rainy season extending from June to September, while the short rainy season extends from March to April. The minimum and maximum annual rainfalls are 834 mm and 1300 mm, respectively [17].

### 2.2. The Population and Study Animals

All lactating cows, either managed by dairy farm owners or smallholders, were the study population. There are 20 dairy farms with formal registration in Holeta town. The number of smallholders identified in the area was not documented, although it is a large population. The dairy farms' herd sizes ranged from 7 to 315 cattle, with 2 to 152 lactating cows. Overall, 630 lactating cows were identified in the study area during the study period.

### 2.3. Study Design

A cross-sectional study design was undertaken from December 2018 to October 2019 to estimate the prevalence, associated risk factors for contamination, load, and antibiograms of *S. aureus* in milk and milk products in Holeta town, central Ethiopia.

### 2.4. Sample Size Determination and Sampling Technique

The sample size was estimated following the method described by Thrusfield [18] for simple random sampling with 95% confidence interval, 5% absolute precision, and an expected prevalence of 47% for *Staphylococcus aureus* in milk in Ethiopia [13]. *N* = *Z*^2^*P*_exp_ (1 − *P*_exp_)/*D*^2^, where *Z* = 1.96, *N* = sample size, *P*_exp_ = expected prevalence, and *D* = absolute precision. Accordingly, 383 samples of milk were collected. In addition to the raw milk samples, 34 bulk tank milk samples from farms, 13 bulk tank milk samples from collection centers, 29 curd milk samples (*Ergo*), and 28 samples of Ethiopian cottage cheese (*Ayib*) were included in the study. Overall, 486 samples were considered in the study. The milk samples were collected from 34 herds. Bulk tank milk samples were collected from farms and collection centers, curd milk (*Ergo*), and cottage cheese (*Ayib*) samples were purchased from restaurants, hotels, and markets.

### 2.5. Sample Collection and Transportation

The milk samples were taken from lactating dairy cows according to an earlier protocol [19]. Briefly, the quarters were washed with tap water and dried. Then, after discarding the first two streams of milk, 10–15 ml milk was collected aseptically into a prelabelled sterile test tube. A sample of bulk tank milk at the farm was taken after milking was completed, and the milk of all cows was mixed in a milk container. While the samples of bulk tank milk at collection centers were collected after the milk fetched by several people from different sites was gathered and mixed. After thorough or full homogenization, milk samples from tanks were eventually collected. Before anyone entered the farm to buy raw milk, milk samples from the bulk tank were gathered. The hygiene of milk containers was mostly unsatisfactory, whether on the farm or at the milk collection site. From the sampling places, 100 ml of curd milk and 100 g of cottage cheese were collected in a sterile universal bottle and maintained at 4°C. Finally, samples were held in an icebox with ice packs for transportation to Ambo University Zoonotic and Food Safety Research Laboratory for isolation and identification of *Staphylococcus aureus*. The samples were immediately cultured or stored at 4°C for a maximum of 24 hours, until cultured on standard bacteriological media.

### 2.6. Enumeration of *Staphylococcus aureus* in Milk and Bulk Tank Milk

Enumeration of *S. aureus* from raw milk samples was performed according to ISO 6888–1:1999 +A1:2003 guidelines protocol [20]. Briefly, 1 ml of udder and bulk tank milk was homogenized into 9 ml of serial peptone water. Then, serial dilutions were prepared. From the 10-fold dilutions of the homogenized, 0.5 ml of 10–3, 10–4, 10–4, and 10–6 dilutions were cultured on Baird Parker Agar Base (Sisco, India) supplemented with egg yolk emulsion and potassium tellurite (England, Basingstoke) using the spread method.

The plates were then incubated at 37°C for 24–48 hrs. Black, glossy, and convex colonies with a diameter of 1–1.5 mm were considered *Staphylococcus aureus* for counting using the colony counter. The counts for each plate were expressed as colony-forming units of the suspension (CFU/ml). Plates that contained 20–200 colonies were selected for *S. aureus* count, and total *S. aureus* colonies from two consecutive plates of each sample were converted into colony-forming units per ml (CFU/ml) using a formula given by Public Health England [21].

(1) $$\begin{array}{c} N=\frac{\sum a}{V\left(n_{1}+n_{2}\right)d}, \end{array}$$

where *N* is the number of bacterial colonies counted, ∑*a* is the sum identified in two consecutive dilution steps, where at least one contained 20 colonies and less than 200 colonies, *n*_1_ is the number of plates counted at the first dilution, *n*_2_ is the number of plates counted at the second dilution, and *d* the dilution rate corresponding to the first dilution selected (initial suspension is a dilution).

### 2.7. Isolation and Identification of *S. aureus*

Isolation and identification of *S. aureus* from milk and milk products were performed following the procedures of ISO (ISO-6888/1/1999) [22]. The tests performed to identify the *S. aureus* isolates included growth characteristics on blood agar, Gram staining, catalase test, growth on Mannitol salt agar base, slide and tube coagulase tests, and growth on purple agar base.

### 2.8. Antimicrobial Susceptibility Test

The *S. aureus* isolates (*n* = 40), which were randomly selected from 52 isolates, were subjected to an antimicrobial susceptibility test against 12 commercially available antimicrobial discs (Oxoid, UK) selected based on common usage [23]. Out of the 40 randomly selected *Staphylococcus aureus* isolates subjected to antimicrobial susceptibility testing, 22, 8, 6, and 4 samples were from raw udder milk, bulk tank milk, curdle milk, and ayib (cottage cheese), respectively. The antimicrobial discs used include vancomycin (30 *μ*g), tetracycline (30 *μ*g), chloramphenicol (30 *μ* g), amoxycillin (2 *μ*g), norfloxacin (10 *μ*g), nitrofurantoin (300 *μ*g), gentamycin (10 *μ*g), cefotaxime (30 *μ*g), ampicillin (10 *μ*g), oxacillin (1 *μ*g), nalidixic acid (30 *μ*g), and azithromycin (15 *μ*g). An antimicrobial susceptibility test was conducted using the Kirby-–Bauer disc diffusion method following the guidelines established by the Clinical and Laboratory Standards Institute [24]. Two to three pure fresh colonies of the isolates from nutrient agar were used to prepare a cell suspension in nutrient broth (HiMedia, India) and incubated for 4–6 hrs at 37°C. Following this, the cell suspension turbidity was attuned equal to 0.5 McFarland standard. Then, a sterile cotton swab was used to spread the bacterial suspension on the Muller Hinton agar (HiMedia, India). The discs were firmly placed in the interval of 3 cm spacing from each other onto the medium with sterile forceps and then incubated at 37°C for 24 hrs. Then, the diameter of clear zones around the discs was measured with a ruler against a black background and compared with standards given by CLSI [23, 25]. *S. aureus* isolates resistant to three and above antimicrobial classes were considered multidrug-resistant.

### 2.9. Questionnaire Survey

A pretested structured questionnaire was used to gather information on potential factors for *Staphylococcus aureus* contamination of milk and milk products. The risk factors considered were cow age (≤5, >5), breed (Holstein Friesian Cross, Jersey), parities (1-2, 3–5), lactation stages (Early ((1-2 months), mid (3–6 months) and late (>7 months)), milking utensils (plastic, stainless steel), teat washing (yes or no), towel use (yes or no), milking techniques (machine or manual), teat washing (yes or no), individual towel use (yes or no), farm size (small (≤10 and large (>10), herd size (<30, ≥30 animals), management system (intensive and semi-intensive), and tick infestation (yes or no). Data on potential risk factors were collected from the interview of owners and observations. In addition, observational checklists were used to rate the hygiene of milk and milk product utensils (poor-undesirable smell and unclean, moderate), and in farm hygiene (poor-gross dirt and smell, moderate).

### 2.10. Data Management and Analysis

The data were entered into Microsoft Excel Spreadsheet 2021, and STATA version 14.2 software (Stata Corp., College Station, USA) was used to analyze it. Descriptive statistics were used to summarize the prevalence of the infection and antimicrobial susceptibility data. Pearson's chi-square or Fisher's exact test was used to analyze the association of categorical variables. Univariable and multivariable logistic regression analyses were performed to assess the association between the prevalence of *S. aureus* and potential risk factors in raw cow milk. For the multivariable model, noncollinear variables with a *P* value of less than 0.25 in the univariable analysis were selected. The *S. aureus* count data/ml of milk was first transformed to the logarithm of base ten (log counts/ml) before analysis. One-way analysis of variance (ANOVA) including the Bonferroni post hoc test was employed to assess the association between *S. aureus* count data and independent variables (sample source, storing milk in the refrigerator, and freshness of milk). The results were considered significant at *P* < 0.05 at all levels of analysis.

## 3. Results

### 3.1. Prevalence of *S*. *aureus*

Out of the total 486 samples examined, 10.69% (52/486) showed the occurrence of *S. aureus*. The prevalence of *S. aureus* in different sample types is summarized in Table 1.

### 3.2. Risk Factors Associated with *S*. *aureus* Occurrence in Milk and Milk Products

In this study, 47 farm owners including milk collection centers were interviewed. A high percentage of the respondents used cold water and soap, followed by hot water for cleaning milk cans (utensils). The majority of farms (85.11%) utilized plastic containers that had previously been used for paint, although 14.9% used stainless steel milk containers. This study indicated that the prevalence of *S. aureus* isolated from milking utensils with poor hygiene was higher than that of milking utensils with moderate hygiene. The potential risk factors associated with the occurrence of *S. aureus* in bulk tank milk are presented in Table 2.

A lower prevalence of *S. aureus* was found in semi-intensively managed farms compared to intensively managed farms. Concerning housing types, a high prevalence of *S. aureus* was found in cattle housed individually compared to cows kept in loose housing. Most farms use common disinfectants (ethanol and Savlon) to clean their hands and equipment after completing their work. The prevalence of *S. aureus* with potential risk factors at the farm level is summarized in Table 3.

The prevalence of *S. aureus* in milk products was significantly higher (*P* < 0.05) in using containers whose hygiene is poor than moderate and in milk products handled by personnel with long nails, unclean, and decorated hands than those with short nails, and clean and nondecorated hands. All respondents reported the use of plastic containers for handling milk products. Similarly, all respondents also reported that they have the habit of fingering the nose, believe that human beings release microorganisms into the surroundings while sneezing and talking, and did not have the behavior of washing hands after handling currency. The prevalence of *S. aureus* and its association with the independent variables studied are presented in Table 4.

The current investigation revealed that the rate of isolation of *S. aureus* was significantly high in curd milk than in other types of samples (*P* = 0.010). The likelihood of the occurrence of *S. aureus* in curd milk was three times higher than that in raw milk (Table 1). Tick infestation was significantly associated with the occurrence of *S. aureus* in raw milk. Parity, stage of lactation, teat washing, use of individual towel, and tick infestation were the variables that were noncollinear with each other, had a univariable *P* < 0.25, and hence entered into the multivariable model. The multivariable logistic regression analysis showed that tick infestation, teat washing, and towel use were significantly associated with *S. aureus* occurrence (Table 5). The likelihood of isolation of *S. aureus* from raw milk was 4.31 times higher in cows infested with ticks compared to those without ticks.

### 3.3. Enumeration of *Staphylococcus aureus*

The current investigation showed that the maximum of 6.92 × 10^7^ CFU/ml and 5.09 × 10^5^ CFU/ml *S. aureus* loads was observed in bulk tank milk collected from the bucket in the farm and raw milk, respectively. Additionally, 6.54 × 10^6^ CFU/ml and 4.36 × 10^7^ CFU/ml of *S. aureus* were counted from bulk milk collected in milk collection centers and restaurants, respectively. In this study, the mean count of *S. aureus* load in raw milk (udder milk) was 4.24 [±1.03]. The count of *S. aureus* was not significantly different concerning sample type (udder milk, bulk tank milk) (*F* = 1.41, *P* = 0.2566), use of refrigerator (*F* = 0.33, *P* = 0.5770), and freshness of milk (*F* = 0.18, *P* = 0.6808).

This study showed that 15 of the 31 samples examined (48.39%) had *S. aureus* count higher than 10^5^ CFU/ml, which was much higher than the level recommended for human consumption (>20 CFU/ml).  Table 6 shows the load of *S. aureus* in milk and milk products.

### 3.4. Antimicrobial Susceptibility Test

*S. aureus* isolates showed alarming levels of resistance to commonly used antimicrobial drugs for veterinary and human health. *S. aureus* showed high “in vitro resistance” to antibiotics such as ampicillin (95%), amoxicillin (95%), oxacillin (87.5%), and cefotaxime (80%) (Table 7).

#### 3.4.1. Multidrug Resistance

In this study, 35% (14/40) of the isolates showed resistance to two antimicrobial classes, while 65% (26/40) of the isolates showed MDR. All isolates are resistant to at least two classes of antimicrobial drugs. The highest multiple drug resistance (MDR) noted was from isolates of raw milk (raw milk) (76.20%) and bulk tank milk (62.5%). Four isolates from raw udder milk showed intermediate susceptibility (19.05%). Three of the eight (37.5%) and five of the eight (62.5%) isolates from bulk tank milk showed resistance to 2 and ≥3 classes of antimicrobial drugs. Similarly, three of the four (75.0%), and one of the four (25.0%) isolates from cottage cheese showed resistance to 2 and ≥3 classes of antimicrobial drugs. Of the seven curd milk isolates tested, 3 (42.86%) and 4 (57.14%) isolates showed resistance to two and ≥3 classes of antimicrobial drugs. The maximum multiple drug resistance registered was resistance to six classes of antimicrobials. penicillin, quinolones, tetracycline, cephems, and aminoglycosides were the most frequent antimicrobial classes where multidrug resistance was observed. Several isolates showed resistance to ampicillin, cefotaxime, oxacillin, amoxicillin, and tetracycline.

The drug resistance patterns of *S. aureus* are presented in Table 8.

## 4. Discussion

The current investigation found an overall 10.69% prevalence of *S. aureus* in milk and milk products, which was in accord with the reports from Holeta, Ethiopia (13.8%) [26], Malaysia (12.4%) [27], Asella (14.9%) [28], Italy (12.9%) [29], and Gujarat, India (10.16%) [30]. The current figure, however, was greater than that of Iran (5.8%) [31] and China (8.2%) [32]. A higher prevalence of *S. aureus* than in the current study was also reported, ranging from 19.6 to 47% in dairy farms in Holeta town [13], Hawasa area [14], Oromia Regional State [15], Tigray region [33], Central Ethiopia [34], Sebeta, Ethiopia [35], South-West Uganda [36], and North-Central and North-Eastern Greece [37]. This variability in the prevalence of *S. aureus* among various studies could be due to the differences in geographical location, management systems, sample size, and hygienic practices employed in farms and milk collection centers. The use of screening tests such as the California Mastitis Test, which identifies positive samples for further culturing, improves the chances of detecting S. aureus in milk and milk products.

The prevalence of *S. aureus* in curd milk was higher in this study (24.14%), which is consistent with the 25.4% reported from the Tigray region, Ethiopia [33], and 21.1% in north-central and north-eastern Greece [37]. However, the current study contradicts the findings of South-West Uganda (12.1%) [36], Iran (0.00%) [31], and Annand, Gujarat (3.33%) [38]. Ethiopian cottage cheese and curd milk are the major milk products produced in the study area. The high prevalence of *S. aureus* in curd milk indicates tolerance of these bacteria to lactic acid produced by competent bacteria. Improving food handlers' and equipment hygiene, as well as the application of cold chain facilities, was required in the milk chain to protect the consumer from milk-borne hazards [33]. Controlling *S. aureus* in dairy products is needed for commercial and profitable small-scale cow farming to improve milk quality for consumers as well as dairy industries.

In this study, 16.78% of respondents were observed to wash cow udders before milking. This finding disagrees with various reports [39–41] who reported 28.21–58.9% of the respondents washing their udders before milking. *S. aureus* is usually found on the udder or teat surface of infected cows and is the primary source of infection between uninfected and infected udder quarters, usually during milking [39]. Milkers did not use the glove, which is considered an important tool for the prevention of the spread of contagious pathogens from cow to cow [42]. Poor hygiene during milking could increase the risk of intramammary infection by *S. aureus* [43]. Postmilking liner contamination by *S. aureus*, seen after the milking of most of the cows, originated from the teat skin and teat canals of healthy cows [44]. The main source of infection is the udder of infected cows that transfer pathogens via the milker's hands, utensils, towels, and the environment (floor) in which the cows are kept [45]. People working in dairy farms were one of the important risk factors that enhanced the contamination with *S. aureus*. Thus, it is important that milkers adequately wash their hands before milking cows [39].

In this study, 31.58% of *S*. *aureus* was isolated from the raw milk of cows infested with ticks around the udder and perineal region. This result was lower compared to the findings of [46] who reported that 63.5% of tick-infested dairy cows shed microorganisms in the milk and were positive for mastitis. Ticks spread pathogens from one animal to another. They create a suitable environment to aid microbial pathogenesis. Most studies have reported a higher prevalence of mastitis in cases where ticks were infected. Tick infestation serves as a source of bacterial transmission from one animal to another, especially contagious pathogens like *S. aureus* [46].

In the current study, the prevalence of *S. aureus* in intensive management systems was 40.91%, which is relatively low compared to the findings of [47] who reported a prevalence of 52.9% but higher than the 10.5% prevalence of *S. aureus* reported by [48]. The high prevalence in intensively managed cows might be due to the keeping of cows in dirty and muddy common barns without bedding materials and failing to use separate towels for individual cows [39]. This could lead to a high chance of contamination of the udder and milk with pathogenic microorganisms. *S. aureus* has adapted to survive in the udder, known for its contagious nature, and is shed in the milk, which serves as a source of infection for other healthy cows during the milking process. It is generally observed that large herds, often managed intensively, are characterized by increased stocking density and increased risk of exposure to infection [45].

In this study, farm size was significantly associated (*P* < 0.05) with the occurrence of *S. aureus*. The prevalence of *S. aureus* in large-scale dairy farms (68.00%) was lower than the reports from Minnesota (84%) [49] and from in and around Asella town, Ethiopia (76.19%) [50]. However, the current result was higher than the reports in China (12.2% [51] and 19.8% [52]) from large-scale farms. Having more cows in a herd infected with the *S. aureus* pathogen would serve to increase the infectious pressure on the quarters, making it more likely for them to acquire an intramammary infection [53]. Biosecurity and management practices should be strictly implemented within farms to prevent the spread of the infection [54]. The high prevalence of *Staphylococcus aureus* in dairy cattle farms might be associated with hygienic and management factors such as breed, farm size, absence of teat dipping practice before and after milking, lack of diagnosing subclinical and chronic forms of mastitis, absence of dry cow therapy, and diagnostic facilities, and practice of hand milking in the dairy farms [51]. Milking infected cows at the end of the milking session and, using a separate milking unit on these cows, especially in herds where multiple employees are involved in the milking process, was difficult, and this may increase the prevalence of *S. aureus* in large farms.

A high proportion of milk product handlers (42.31%) during the study period had long nails, wore jewelry, and had decorated hands. These results are similar to the study conducted in Sebeta and Arsi Nagelle (31.8%) [35] but higher than the 18.18% [55] and 3.3% [8] reported from milkers' hands and food handlers in Japan and Sao Paolo, Brazil, respectively. The colonization of *S. aureus* in different food handlers' noses and hands suggested possible transmission and potential risk of milk product contamination during handling and transportation. Milk handlers and milk buckets could be potential sources of contamination of milk with *S. aureus* [35].

In this study, the hygiene of milk product containers was significantly associated with the occurrence of *S. aureus* due to the poor-quality milk product containers used (30.77%). The prolonged use of poor-quality plastic materials for handling milk products was common in milk collection centers and among farmers presenting milk products to the markets. Utensils used for milking and storage determine the safety of milk and milk products [48]. This could be explained by the proliferation of *S. aureus* due to heat, their ability to form biofilm in milk product containers, and their resistance to insufficient cleaning. Milk and milk products can be contaminated after heat treatment due to poor hygiene of milk product containers, and the main sources of contamination are infected food handlers, in addition to infections of animal origin [29]. Equipment used for milking, collecting, and storage determines the quality of milk and milk products [56]. Frequent use of milk product containers without enough cleaning may increase contamination of the product by *S. aureus*. The use of plastic and traditional containers (clay pots) can be a potential source for the contamination of milk because they allow the multiplication of bacteria on milk contact surfaces during the interval between milking processes. *S. aureus* persists and proliferates in milk buckets due to heat, their ability to form biofilm in collecting and storage tanks, and their resistance to insufficient cleaning [57]. The main reasons for the high prevalence of *S. aureus* are a lack of implementation of routine food-borne pathogen prevention and control practices by farms, milk collection centers, and milk product handlers, as well as the dominance of risk factors identified in this study.

The high prevalence of *S. aureus* in milk products handled by respondents who frequently wipe hands using dirty clothes with a possible high load of microorganisms suggests the potential carryover of *S. aureus* to milk products.

The significantly high isolation rates of *S. aureus* in which milkers use individual cow towels (47.06%) and among cows whose udder was washed were not to our expectations and contradicted most published information. Nevertheless, from visual observation during sample collection, the water that milkers used for cleaning towels and their hands was from a single container and was not changed during milking of all cows. Thus, in the study area, teat washing exacerbates *S. aureus* prevalence rather than minimizing it due to the use of contaminated water and towels. Cross-contamination of *S. aureus* may occur via the repeated use of clothes for wiping different teats of the same cow, transfer of pathogens via the dip cup used between teats and between cows, or milking machine contamination [53]. Teat dipping and the use of a single towel per cow can be important, as *S. aureus* teat colonization can be significantly associated with *S. aureus* intramammary infection (IMI) [58]. Since drying was not practiced sufficiently by the cow milkers in the study area, the contamination level of the milk is expected to be high. The predominant source of infection is the udder of infected cows transmitted through the milker's hands, utensils, towels, and the environment (floor) in which the cows are kept [45]. *S. aureus* is extremely resistant to environmental stresses, surviving temperature, and moisture extremes [15].

Even though equipment, udder, teat, milkers' hygiene, and good milk handling practices are very essential to reduce contamination of milk by *S. aureus* as well as subsequent public health risks, in this study these points are inadequately implemented.

In the current study, the total *S. aureus* count in each *Staphylococcus aureus* positive raw milk and bulk tank milk sample was above 10^5^ CFU/ml. Based on the standard level ISO 6888 [22], such milk is unsatisfactory, and if consumed, it might constitute a serious risk to the health of the population. When the concentration of an enterotoxigenic strain of *S. aureus* exceeds 10^5^ CFU/ml, the strain is capable of releasing sufficient enterotoxin [21, 49].

In the current study, high resistance of *S. aureus* to ampicillin (95%) and amoxicillin (95%) followed by oxacillin (87.5%) and cefotaxime (80%) was observed. The current investigation was in harmony with 94.3–100% resistance of *S. aureus* to ampicillin reported from dairy cow milk in China and Ambo [59, 60]. On the other hand, lower resistance to ampicillin ranging from 33.33% to 67.9% has been previously reported [14, 30, 36, 38]. In contrast to the present findings, low resistance to amoxicillin ranging from 30.8% to 68.29% has been previously reported [14, 15, 36, 61].

Resistance to ampicillin and amoxicillin is not surprising because these drugs are the most commonly used antimicrobials for the treatment of infections in humans and veterinary practice for many years in Ethiopia [47]. The extensive use of antimicrobials in dairy animals has partly increased the emergence of antimicrobial resistance. The resistance of *S. aureus* to amoxicillin and ampicillin may be attributed to the production of beta-lactamase, an enzyme that inactivates penicillin and closely related antibiotics [60]. The resistance of *S. aureus* strains to oxacillin in the present study (13.6%) was lower than in the previous reports (60.3%) [14, 27, 38, 50, 60].

In the current study, *S. aureus* isolates showed 32.5% resistance to tetracycline, which was lower compared to the high resistance (40%–82.2%) previously reported from different sources [27, 46, 47, 50, 62]. In contrast to the present findings, Sharma et al. [63] reported that several of their *S*. *aureus* isolates were susceptible to ampicillin, tetracycline, and oxacillin. The variability in resistance results could partly arise from how frequently the drug was used in the study area.

The present study revealed that 62.5% of *S. aureus* tested were multidrug-resistant (MDR). The antimicrobial susceptibility tests revealed that the isolates had the characteristics of a general multidrug resistance pattern (ampicillin, amoxicillin, oxacillin, cefotaxime, and tetracycline). This is comparable with the findings of [63] who reported a higher prevalence of multidrug-resistant *S. aureus* (60–70%) in raw milk of dairy cattle in India. The emergence of resistance to many drugs represents a public health hazard because food-borne outbreaks might be difficult to treat and the group of MDR *S. aureus* in the food supply represents a reservoir for communicable resistant genes [64]. This could be attributed to the erratic and extensive use of antibacterial drugs without prior antimicrobial susceptibility testing. Such antimicrobial-resistant organisms can pose serious health-related hazards to animals as well as human beings. Currently, an increasing antimicrobial resistance rate has been reported in *S. aureus* from bovine mastitis [32, 63].

A limitation of this study is that environmental samples and personnel were not sampled due to the scarcity of facilities. The results of milk products should be cautiously interpreted as the sample size might not warrant full generalization of the findings to the surrounding areas. Molecular characterization of enterotoxin genes was not conducted due to a lack of budget and laboratory facilities. Thus, in future studies of this type, it is better to sample farm environments and perform molecular characterization of enterotoxin genes.

## 5. Conclusions

The present study has shown that *Staphylococcus aureus* is widely prevalent in milk and milk products in Holeta town. Teat washing, towel use, and tick infestation are the determinants of *Staphylococcus aureus* milk contamination. In addition, *S. aureus* variably occurs on different contact surfaces that have close contact with the milk production process. The high rate of isolation and the high load of *S. aureus,* which did not comply with the current standard, indicates the higher public health risk due to the widespread consumption of raw milk and its products in Ethiopia. The results also emphasize the importance of regular microbiological examination of milk and milk products for the production of quality and safe products. Moreover, the large proportion of MDR *S. aureus* isolates may impede effective control of *S. aureus* udder infection in cows as well as present a public health risk due to the spread of drug-resistant zoonotic *S. aureus*. Antiseptics and disinfectants should be encouraged after washing hands and cleaning milk utensils, respectively. Educational programs to increase knowledge and raise awareness of farmworkers, milk product handlers, and milk collection centers on the importance of good hygiene help to increase the good practices of food handlers, which could significantly reduce contamination levels. Routine spraying of animals with acaricide should be performed to control tick infestation. Rational use of antimicrobial drugs and regular surveillance of antimicrobial resistance should be made to combat drug resistance.

## Figures and Tables

**Table 1:** Univariable logistic regression analysis of *S. aureus* prevalence in different sample types.

Sample type	No. of tested	No. of positive	% prevalence (95% CI)	OR (95% CI)	*P* value
Raw milk	383	33	8.64 (6.02–11.91)	1 (−)	—
Bulk tank milk at farm	34	5	14.73 (4.95–31.05)	1.82 (0.66–5.03)	0.246
Bulk tank milk at collection centers	13	3	23.08 (5.04–53.81)	3.17 (0.83–12.10)	0.091
Cottage cheese	28	4	14.29 (4.03–32.66)	1.76 (0.57–5.38)	0.320
Curd milk	29	7	24.14 (10.30–43.54)	3.36 (1.34–8.46)	0.010
Overall	486	52	10.69 (8.09–13.79)		

No. = number, CI = confidence interval, OR = odd ratio, chi-square (*X*^2^) = 0.21, and *P* value = 0.037.

**Table 2:** Results of the association between the prevalence of *Staphylococcus aureus* and potential risk factors in bulk tank milk.

Variables	Categories	No. of tested	No. of positive	Percentage	Fisher's exact test *P* value
How to clean bulk tank container	Cold water and soap	38	4	10.53	0.033
	Hot water	9	4	44.44	

Types of milking utensils	Plastic	40	6	15.00	0.585
	Stainless steel	7	2	28.57	

Hygiene of milking utensils	Moderate	28	2	7.14	0.047
	Poor	19	6	31.58	

**Table 3:** Results of the analysis of the association between the prevalence of *Staphylococcus aureus* and potential risk factors in dairy cattle farms.

Variables	Categories	No. of tested	No. of positive	Percentage	Chi-square	*P* value
Farm hygiene	Moderate	10	4	40.00	—	0.457^*∗*^
	Poor	24	14	58.33		

Farm size	Small (≤10)	9	2	22.22	—	0.052^*∗*^
	Large (>10)	25	16	64.00		

Management system	Intensive	12	10	83.33	6.88	0.009
	Semi-int.	22	8	36.36		

Food safety training	No	26	11	42.31	5.02	0.025
	Yes	8	7	87.50		

Use of disinfectant	No	27	15	55.56	—	0.681^*∗*^
	Yes	7	3	42.86		

Housing types	Loose	27	12	44.44	3.80	0.051
	Individual	7	6	85.71		

Sanitation of the farm	Poor	20	9	45.00	1.23	0.268
	Fair	14	9	64.29		

Fisher's exact test *P* value; semi-int. = semi-intensive.

**Table 4:** Results of the analysis of the association of the prevalence of *Staphylococcus aureus* in milk products with independent variables.

Variables	Categories	No. of tested	No. of positive	Percent	*P* value
Sample type	Cottage cheese	28	4	14.29	0.504
	Curd milk	29	7	24.14	

Hygiene of the product container	Moderate	31	3	9.68	0.089
	Poor	26	8	30.77	

Long nails, and unclean and decorated hand	No	31	0	0.00	≤0.001
	Yes	26	11	42.31	

Have food safety information	Yes	29	5	17.24	0.747
	No	28	6	21.43	

Wipe hands on dirty clothes	No	34	3	8.82	0.020
	Yes	23	8	34.78	

Agents of food-borne disease are found everywhere	No	28	5	17.86	1.000
	Yes	29	6	20.69	

Microorganisms are present on human skin	No	23	4	17.39	1.000
	Yes	34	7	20.59	

**Table 5:** Results of logistic regression analysis of potential risk factors associated with the prevalence of *Staphylococcus aureus* in raw milk.

Variables	Categories	No. of tested	No. of positive (%)	Univariable		Multivariable	
				OR (95% CI)	*P* value	OR (95% CI)	*P* value
Age (years)	≤5	161	12 (7.45)	1.0	—		
	>5	221	21 (9.5)	1.03 (0.62–2.73)	0.483		

Breed	HF cross	325	27 (8.31)	1.0	—		
	Jersey	57	6 (10.53)	1.29 (0.51–3.30)	0.583		

Parity	3–5	46	1 (2.17)	1.0	—	1.0	
	>6	156	12 (6.79)	3.75 (0.47–29.64)	0.210	2.83 (0.32–25.18)	0.351
	1-2	180	20 (11.11)	5.62 (0.73–43.06)	0.096	6.60 (0.76–57.09)	0.086

Herd size	≥30 animals	215	18 (8.37)	1.0	—		
	<30 animals	167	15 (8.98)	1.08 (0.53–2.21)	0.833		

Farm hygiene	Fair	226	18 (7.96)	1.0	—		
	Poor	99	9 (9.09)	1.16 (0.50–2.67)	0.735		
	Good	57	6 (10.53)	1.36 (0.51–3.60)	0.536		

Management system	Intensive	254	20 (7.81)	1.0			
	Semi-intensive	128	13 (10.16)	1.32 (0.63–2.75)	0.457		

Teat status	All normal	341	29 (8.50)	1.0	—		
	At least one is blind	41	4 (9.76)	1.16 90.39–3.49)	0.788		

Lactation stage	Mid (3–6 months)	130	7 (5.38)	1.0	—	1.0	—
	Late (>7 months)	98	10 (10.20)	2.0 (0.73–5.45)	0.177	1.94 (0.65–5.79)	0.236
	Early (1-2 months)	154	16 (10.39)	2.04 (0.81–5.12)	0.130	1.79 (0.65–4.87)	0.257

Milking utensils	Stainless steel	227	19 (8.37)	1.0	—		
	Plastic	155	14 (9.03)	1.08 (0.53–2.24)	0.821		

Method of cleaning milking containers	Hot water and detergent	58	4 (6.90)	1.0	—		
	Cold water and soap/detergent	324	29 (8.95)	1.33 (0.45–3.93)	0.609		

Use of disinfection	Yes	258	22 (8.53)	1.0	—		
	No	124	11 (8.87)	1.04 (0.49–2.23)	0.911		

The habit of fingering nose	No	24	1 (4.17)	1.0	—		
	Yes	358	32 (8.94)	2.56 (0.30–17.27)	0.433		

Teat washing	No	233	8 (3.43)	1.0	—	1.0	—
	Yes	149	25 (16.78)	5.67 (2.48–12.94)	≤0.001	4.93 (2.06–11.81)	≤0.001

Individual towel use	No	365	25 (6.85)	1.0		1.0	
	Yes	17	8 (47.06)	12.09 (4.29–34.04)	0.001	12.13 (3.74–39.29)	≤0.001

Milking technique	Machine	106	8 (7.55)	1.0	—		
	Manual	276	25 (9.06)	1.20 (0.52–2.76)	0.663		

Tick infestation	No	363	27 (7.44)	1.0	—	1.0	
	Yes	19	6 (31.57)	5.74 (2.02–16.31)	0.001	4.31 (1.28–14.44)	0.018

CI = confidence interval; OR = odds ratio.

**Table 6:** *S. aureus* counts in log10 CFU/ml by sample source, sample type, and storage condition.

ID of cont. samples	Source of samples	Sample type	Storage in refrigerator	*S. aureus* count (CFU/ml)	*S. aureus* log 10 CFU/ml
AB-405	Farm	Udder milk	Yes	3.16 × 10^7^	7.500187
AB-408	Farm	Bulk tank	Yes	6.92 × 10^7^	7.840562
AB-409	Restaurant	Bulk tank	Yes	3.85 × 10^6^	6.585973
AB-412	Farm	Udder milk	Yes	5.69 × 10^6^	6.755182
AB-413	Farm	Bulk tank	Yes	2.61 × 10^7^	7.418
AB-420	Farm	Udder milk	Yes	5.09 × 10^5^	5.706795
AB-421	Farm	Bulk tank	Yes	5.96 × 10^7^	7.775511
AB-422	Restaurant	Udder milk	Yes	2.76 × 10^5^	5.441481
AB-423	Restaurant	Bulk tank	Yes	4.36 × 10^7^	7.639849
AB-424	Restaurant	Bulk tank	Yes	2.09 × 10^5^	5.320335
AB-426	Farm	Udder milk	No	5.20 × 10^7^	7.716003
AB-429	Restaurant	Bulk tank	Yes	2.43 × 10^7^	7.386742
AB-468	Milk collection centers	Udder milk	No	6.54 × 10^6^	6.81594
AB-469	Milk collection centers	Udder milk	Yes	4.81 × 10^6^	6.682883
AB-472	Milk collection centers	Bulk tank	Yes	2.83 × 10^7^	7.452762

**Table 7:** Antimicrobial resistance profiles of *S. aureus* from milk and milk products (*n* = 40).

Classes of antimicrobial agents	Antimicrobials	Disc conc. (*μ*g)	Susceptible	Intermediate	Resistance
			No. (%)	No. (%)	No. (%)
Glycopeptides	Vancomycin	30	28 (70.00)	8 (20.00)	4 (10.00)
Tetracyclines	Tetracycline	30	27 (67.50)	—	13 (32.50)
Phenicols	Chloramphenicol	30	37 (92.50)	—	3 (7.50)
Quinolones	Nalidixic acid	30	10 (25.00)	15 (37.50)	15 (37.50)
Fluoroquinolones	Norfloxacin	10	37 (92.50)	—	3 (7.50)
Nitrofurantoin	Nitrofurantoin	300	36 (90.00)	2 (5.00)	2 (5.00)
Aminoglycosides	Gentamycin	10	34 (85.00)	—	6 (15.00)
Cephems	Cefotaxime	30	4 (10.00)	4 (10.00)	32 (80.00)
Penicillin	Ampicillin	10	2 (5.00)	—	38 (95.00)
	Oxacillin	1	5 (12.50)	—	35 (87.50)
	Amoxicillin	2	2 (5.00)	—	38 (95.00)
Macrolides	Azithromycin	15	38 (95.00)	—	2 (5.00)

**Table 8:** Patterns of drug resistance of *S. aureus* isolated from milk and milk products.

Frequencies	Antimicrobial's resistance pattern	No of resistant isolates	Percent
Three	AMX, AMP, GEN	3	7.5
	AMX, AMP, TET	2	5
Total		5	12.5

Four	AMX, AMP, OXA, AZM	1	2.5
	AMX, AMP, OXA, CXT	7	17.5
	AMX, AMP, OXA, TET	2	5
Total		10	25

Five	AMX, AMP, OXA, AZM, CXT	1	2.5
	AMX, AMP, OXA, NAL, CXT	6	15
	AMX, AMP, OXA, TET, CXT	3	7.5
	AMX, AMP, OXA, GEN, CXT	2	5
	AMX, AMP, OXA, VAN, CXT	2	5
	TET, NAL, AMO, NIT, OXA	1	2.5
Total		15	37.5

Six	AMX, AMP, OXA, NIT, CXT, CHL	4	10
	AMX, AMP, OXA, NAL, CXT, NOR AMX, AMP, OXA, NAL, CXT, CHL		
	AMX, AMP, OXA, NAL, CXT, GEN		
Total		4	10

Seven	AMX, AMP, OXA, NAL, CXT, GEN, TET	3	7.5
	AMX, AMP, OXA, NAL, CXT, CHL, TET		
	AMX, AMP, OXA, NAL, CXT, NOR, TET		
Total		3	7.5

Eight	AMX, AMP, OXA, NAL, AZM, GEN, TET, VAN	1	2.5
Total		1	2.5

AMP—ampicillin, AMX—amoxycillin, AZM—azithromycin, CTX—cefotaxime, CHL—chloramphenicol, GEN—gentamicin, NAL—nalidixic acid, NIT—nitrofurantoin, NOR—norfloxacin, OXA—oxacillin, TET—tetracycline, and VAN—vancomycin.

## Data Availability

The authors confirm that all data underlying the findings will be available upon request of the corresponding author fully without restriction.

## Abbreviations

CFU: Colony-forming unit
°C: Degree Celsius
CI: Confidence interval
ISO: International organization for standardization
OR: Odds ratio
*S. aureus*: *Staphylococcus aureus*.
